# Metagenomic analysis of double-stranded DNA viruses in healthy adults

**DOI:** 10.1186/s12915-014-0071-7

**Published:** 2014-09-10

**Authors:** Kristine M Wylie, Kathie A Mihindukulasuriya, Yanjiao Zhou, Erica Sodergren, Gregory A Storch, George M Weinstock

**Affiliations:** The Department of Pediatrics, Washington University School of Medicine, Campus Box 8116, 660 S. Euclid Avenue, St Louis, MO 63110 USA; The Genome Institute, Washington University School of Medicine, Campus Box 8501, 4444 Forest Park Avenue, St Louis, MO 63108 USA; Current address: The Jackson Laboratory for Genomic Medicine, 10 Discovery Drive, Farmington, CT 06032 USA

**Keywords:** Metagenomics, Microbiome, Virome

## Abstract

**Background:**

The Human Microbiome Project (HMP) was undertaken with the goal of defining microbial communities in and on the bodies of healthy individuals using high-throughput metagenomic sequencing analysis. The viruses present in these microbial communities, the ‘human virome,’ are an important aspect of the human microbiome that is particularly understudied in the absence of overt disease. We analyzed eukaryotic double-stranded DNA (dsDNA) viruses, together with dsDNA replicative intermediates of single-stranded DNA viruses, in metagenomic sequence data generated by the HMP. We studied 706 samples from 102 subjects were studied, with each subject sampled at up to five major body habitats: nose, skin, mouth, vagina, and stool. Fifty-one individuals had samples taken at two or three time points 30 to 359 days apart from at least one of the body habitats.

**Results:**

We detected an average of 5.5 viral genera in each individual. At least one virus was detected in 92% of the individuals sampled. These viruses included herpesviruses, papillomaviruses, polyomaviruses, adenoviruses, anelloviruses, parvoviruses, and circoviruses. Each individual had a distinct viral profile, demonstrating the high interpersonal diversity of the virome. Some components of the virome were stable over time.

**Conclusions:**

This study is the first to use high-throughput DNA sequencing to describe the diversity of eukaryotic dsDNA viruses in a large cohort of normal individuals who were sampled at multiple body sites. Our results show that the human virome is a complex component of the microbial flora. Some viruses establish long-term infections that may be associated with increased risk or possibly with protection from disease. A better understanding of the composition and dynamics of the virome may hold important keys to human health.

**Electronic supplementary material:**

The online version of this article (doi:10.1186/s12915-014-0071-7) contains supplementary material, which is available to authorized users.

## Background

The Human Microbiome Project (HMP) was undertaken to define microbial communities found in and on the bodies of healthy individuals [1]. Understanding the range of normal microbial flora will inform the design and interpretation of future studies aimed at associating microbial community states with disease. The HMP has generated the largest, most complex sequence data set from human microbial communities, with greater than seven terabases (about 70 billion sequences) of whole-genome shotgun data generated [2]. Recently the first large-scale analyses of the HMP data were published, focusing exclusively on bacteria [2,3]. Here we present a comprehensive analysis of eukaryotic DNA viruses in the HMP data set.

The viral component of the human microbiome, the human virome, is an important aspect of the HMP. While viruses that cause acute symptomatic infections clearly impact human health, viruses that establish acute or long-term apparently asymptomatic infections are part of the microbial flora and may have unappreciated effects on human health [4]. Describing the characteristics and dynamics of the human virome is a first step in understanding the role of the virome in human health. We report the first large-scale molecular analysis of the viral flora in a cohort of 102 healthy subjects sampled at as many as five major body habitats: nose, skin, mouth, vagina, and stool. Importantly, the analysis was based on the use of high-throughput deep sequencing, allowing the detection of a broad range of DNA viruses, both cultivable and non-cultivable.

## Results

### The human virome in five body habitats in healthy, asymptomatic adults

**Figure 1:**
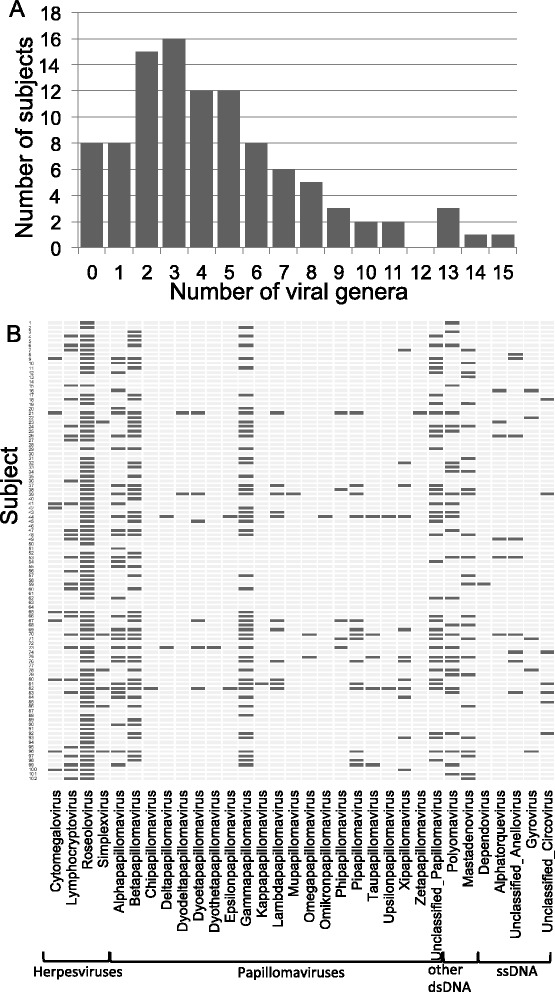
**The human virome in healthy, asymptomatic adults. (A)** The histogram shows the number of individuals (x-axis) who were positive for a given number of different viral genera (y-axis). **(B)** The viral genera (x-axis) detected in each subject (y-axis) are represented by black bars. The virome of each individual is viewed by looking at the black bars in a given row.

**Figure 2:**
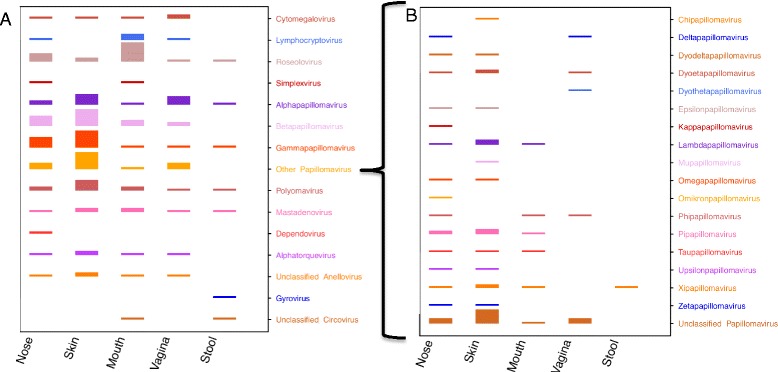
**The human virome in five body habitats. (A)** All of the viruses detected in the five body habitats are shown. Each virus is represented by a colored bar and labeled on the y-axis on the right side. The relative height of the bar reflects the percentage of subjects sampled at each body site in whom the virus was detected. In this panel, the bar representing roseoloviruses in the oral samples reflects the maximum bar height, because 98% of the individuals who were sampled in the mouth harbored roseoloviruses. **(B)** This panel shows papillomaviruses included in the category ‘Other papillomaviruses’. The largest bar height shown represents the unclassified papillomaviruses found in skin samples from 65% of subjects. The percentages represented in this figure are shown in Additional file 1: Table S3.

We detected one to 15 viral genera (average 5.5) in 92% of the 102 individuals sampled (Figure 1A). Figure 1B illustrates the viromes of the 102 individuals defined by sampling up to five major body habitats, showing that a broad range of viruses was detected in healthy people. The 102 individuals carried seven distinct families of human DNA viruses (Figure 2A). The double-stranded DNA (dsDNA) viruses included members of the virus families *Herpesviridae*, *Papillomaviridae*, *Polyomaviridae*, and *Adenoviridae*. In addition to the dsDNA viruses, several genera of single-stranded DNA (ssDNA) viruses were detected, including members of the families *Anelloviridae*, *Parvoviridae*, and *Circoviridae.* These ssDNA viruses generate dsDNA intermediates during replication, which allowed them to be detected with the DNA preparation protocol employed that would not otherwise detect ssDNA viruses. The *Herpesviridae* genera included roseoloviruses (predominantly human herpesvirus (HHV)-7), herpes simplexvirus (HSV-1), lymphocryptovirus (Epstein Barr Virus), and human cytomegalovirus (HHV-5). The papillomaviruses detected were predominantly the most common human papillomaviruses (HPVs): alpha-, beta-, and gamma-papillomaviruses. These included high-risk papillomavirus types, such as HPV-16, HPV-18, and HPV-45. We also detected sequences, predominantly in the nose and skin, with more remote similarity to 17 papillomavirus genera that contain non-human viruses (Figure 2B). This suggests that numerous undescribed HPVs may exist, which is not surprising given that more than 150 HPV genera have been identified [5,6], and new HPVs continue to be discovered [7,8]. Polyomaviruses and adenoviruses were also relatively common. We detected polyomaviruses MWPyV [9], Merkel Cell [10,11], human polyomavirus (HPyV)-6, HPyV7 [12], and JC [13]. The anelloviruses were found in all of the body sites except stool. These viruses establish persistent infections in the blood in the majority of the population early in life [14]. The parvovirus detected was adeno-associated virus, which was detected in conjunction with adenovirus. The gyrovirus and circovirus sequences were found in the oral and gastrointestinal tracts.

Some viruses were found in a particular body site of greater than 30% of the subjects. Roseoloviruses, predominantly HHV-7 and to a lesser extent HHV-6, were present among 98% of the individuals who provided mouth samples, while skin samples collected from the retroauricular crease commonly contained a variety of alpha-, beta- and gamma-papillomaviruses (41%, 76%, and 76% of subjects, respectively). We detected beta- and gamma-papillomaviruses in the anterior nares of 48% of individuals, and roseoloviruses in 33%. The vagina was dominated by papillomaviruses, with 38% of those sampled carrying one or more alpha-papillomaviruses.

The same viruses were prevalent in multiple body habitats within individuals, and these shared components of the individual’s virome were consistent with anatomic or functional links between these body sites. For instance, the beta- and gamma-papillomaviruses were the viruses most commonly found in the skin and the nose (anterior nares; Figure 2A,B), which may reflect proximity and similarities in microenvironments that support infection with these viruses. Roseoloviruses were common in mouth samples, and they were also common in skin and nose habitats (Figure 2A), which could result from transfer of material carrying these viruses. While viruses were detected in few stool samples, most components of the stool virome were shared with the mouth (Figure 2A), for example roseoloviruses and circoviruses, possibly reflecting the passage of oral viruses into the gut.

### Viral profiles of individuals

**Figure 3:**
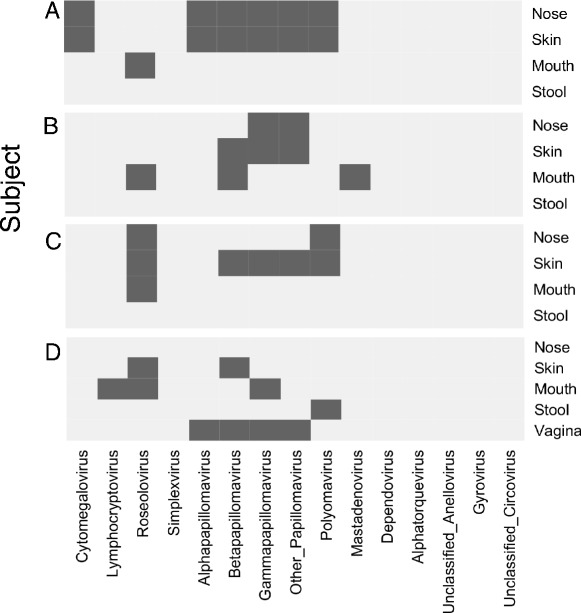
**Individuals have unique viral fingerprints. (A-D)** Each panel represents the virome of a specific subject. The dark rectangles represent the presence of a particular virus (x-axis) in a body habitat (y-axis).

Analysis on an individual level revealed distinct viral profiles and also supported the observation that some viruses are prevalent in multiple body habitats (Figure 1B). We describe a few individual cases here. Figure 3A shows an example in which numerous viruses were shared in the nose and skin samples from one individual, including human cytomegalovirus, several papillomaviruses including HPV-19, and MWPyV. Human adenoviruses were relatively common in oral and skin samples (17% of samples, including the subject in Figure 3B), with nearly all sequences mapping to human adenovirus C reference genomes. Figure 3C shows that roseolovirus HHV-7 was detected in samples from the nose, skin, and mouth of another individual. Some of the virome variation between individuals was found in sequences that lacked nucleotide sequence similarity but shared amino acid sequence similarity with a variety of papillomaviruses, which suggests the presence of novel human papillomavirus types or strains (Figure 3A-D, ‘Other Papillomaviruses’).

### The stability of the human virome

**Figure 4:**
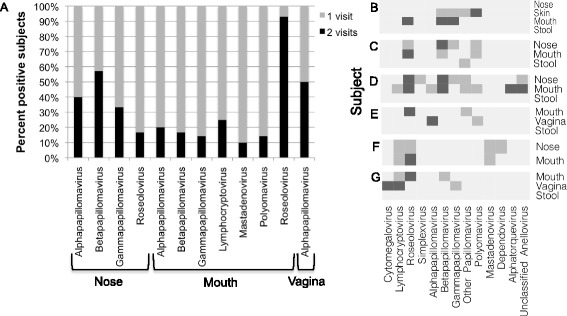
**Stability of the human virome across two evaluations.** The whole genome shotgun sequence data included a subset of samples from 51 individuals, which were collected at two time points. For viruses detected in five or more individuals, Panel **A** shows the percentage of subjects in whom the virus was detected at only one (gray bar) or two consecutive (black bar) visits. The complete census of viruses that were detected in the longitudinal data set is presented in Additional file 1: Table S4. Panels **B-G** display the viruses detected in samples from a subject at one or both evaluations. Gray rectangles signify that the indicated virus was detected in a sample collected in one of two evaluations; black rectangles signify that the indicated virus was detected in the samples collected at both evaluations. Data from six individuals are shown in this figure for the purpose of illustration. Additional file 1: Table S5 shows the data for all subjects with multiple visits.

Fifty-one individuals were sampled on two visits, which allowed us to assess the stability of an individual’s virome (Figure 4 and Additional file 1: Tables S4 and S5). The roseolovirus HHV-7 was commonly detected in samples taken from the same individual on both visits (Figure 4A,B). In fact, if HHV-7 was detected in mouth samples taken during the first evaluation, it was also detected in samples from the second visit in greater than 90% of the subjects. For the individuals in whom alpha-, beta-, and gamma-papillomaviruses were detected in nasal or vaginal samples collected at one evaluation, the virus was also detected in samples from the other visit in 30% to 50% of the subjects (Figure 4A). In samples from the mouth, papillomaviruses were detected less frequently over multiple visits.

### Relationships between bacterial microbiome and virome in the vagina

**Figure 5:**
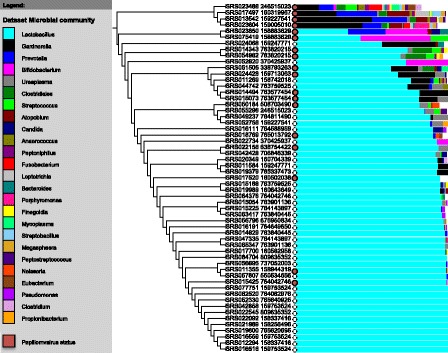
**Variation in the vaginal microbiome.** All posterior fornix samples were clustered based on bacterial community structure. The 20 most abundant components of the bacterial communities are shown in the stacked bar charts. The presence of alpha-papillomaviruses is indicated with a red circle at the base of each stacked bar.

Viruses and bacteria may have dynamic correlative and anti-correlative relationships within the microbiome. Therefore, we analyzed the relationship between the virome of individuals and the composition of their vaginal bacterial microbiome. Alpha-papillomaviruses were detected in 37.5% of subjects who had vaginal samples sequenced. Consistent with previous studies, we detected several types of vaginal bacterial communities [15,16]. Viruses were found in all of these microbial communities, but alpha-papillomaviruses were more common in samples from individuals with more bacterial diversity than those whose vaginal microbiomes were made up almost entirely of *Lactobacillus* (Figure 5). Looking only at the posterior fornix samples from the first visit of each individual (34 samples), subjects with communities containing less than 85% lactobacillus were more likely to have alpha-papillomaviruses in their samples compared with other subjects (*P* = 0.0010, Fisher’s exact test). Subjects carried as many as four distinct alpha-papillomaviruses, including HPV-16, HPV-18, and others that are considered high-risk for tumorigenesis. For example, the vaginal sample from one subject contained HPV-45 (high risk), HPV-53 (probably high risk), and HPV-43 (low risk) [17]. We did not observe other statistically significant correlations of virus sequences with bacteria in other anatomic sites. Nor did we observe statistically significant correlations in other anatomic sites of virus sequences with patient data, such as body mass index, age, gender, or enrollment city (St. Louis, MO or Houston, TX).

## Discussion

Here we report the most comprehensive metagenomic sequence-based analysis of eukaryotic viruses in the human virome to date. This description of the eukaryotic DNA virome is relatively unbiased in that it did not rely on virus culture or virus-specific assays to define the normal viral flora. Previous studies of the human virome have focused primarily on bacteriophages [18-21], which are closely tied to their bacterial hosts and have only recently been shown to have more direct effects on the human habitats they occupy [22]. We demonstrate that healthy humans harbor a surprising variety of DNA viruses that directly infect eukaryotic cells. Some of these viruses appear to be stable components of an individual’s virome. Other components of the virome were detected at a single time point, suggesting these viruses were present transiently or that levels of some viruses in some samples may have fallen beneath the sensitivity of our assay. Future studies aimed at associating DNA viruses with disease in these body habitats will be able to use the data from these healthy individuals to aid interpretation of their results.

While we observed viral nucleic acid sequences in the samples, we have not formally demonstrated that these viruses are replicating. However, we observed some of these viruses consistently in the same subject over multiple visits; we observed viruses in body sites associated with replication and shedding of virus particles rather than latency (HHV-7 in the saliva); and we detected viruses with ssDNA genomes whose dsDNA replication intermediate form could be detected in our sequencing assays (the dependovirus adeno-associated virus). Each of these suggests virus replication. For these reasons, we will use the term ‘infection’ in the discussion of these data, with the understanding that some of the viral sequences detected may represent virus latency or transient presence of viral nucleic acid rather than active infection.

The human body appears to dynamically maintain and control persistent infections, such as HHV-7 and many papillomaviruses. While symptomatic viral infections are currently most well known to medical science, future research is needed to explore the effects of long-term and asymptomatic infections on human health and disease. The virome may confer benefits on the host, the way the bacterial contingent of the microbiome does. For instance, murine herpesvirus infections have been shown to provide protection from infection with other pathogens in mice by up-regulating components of the immune system [23]. Members of the virome may play similar roles in maintaining human health, in a mutualistic relationship with the human host. Evolutionary considerations suggest that the universal presence of a specific microbe may reflect a beneficial interaction with the host. By contrast, some persistent DNA viral infections are clearly linked with disease, as exemplified by the increased risk for cervical cancer in women infected with high-risk HPV strains. Perhaps this is analogous to the persistence of bacterial pathogens in the microbiome that present a potential for disease that is not always realized [24]. The idea that persistent viral infections can protect or predispose the host to other infections is a hypothesis we are pursuing.

Our expanded analysis of eukaryotic DNA viruses of 102 individuals at five major body habitats shows extensive interpersonal variation in virome composition. In addition, we show that although an individual’s virome can change over time, viral presence can be remarkably stable in an individual. Inasmuch as humans are constantly exposed to viruses in the environment, the source of members of the variable virome is not difficult to envision. However, the mechanisms that favor viral-host coexistence likely consist of known protective host defenses and unappreciated processes. Viruses encode proteins to regulate the cell cycle [25], regulate host gene expression, and suppress or subvert host immune responses [26-30]. Viruses also encode micro RNAs that regulate cellular processes [31]. Thus, latent and persistently infecting viruses are continually interacting with the host in many ways. Many virologists are characterizing the complex effects of interactions that occur between virus and host during infection [25-30]. We know that viruses commonly target specific cellular pathways to regulate the cell and promote viral replication, but each virus can have its own mechanism for achieving the same control over a cellular pathway [31,32]. Future studies of the human virome will further address functional interactions between viruses and host cells.

Another important dimension of virome studies is possible interactions between the viral and bacterial communities harbored by complex organisms. To begin to probe this, we studied relationships between eukaryotic viruses and the composition of vaginal bacterial communities. Previous studies defined subtypes of vaginal bacterial microbiomes [15]. Here, using unbiased metagenomic sequencing we observed that alpha-papillomaviruses, which include the known oncogenic papillomavirus types, were more common in women with a bacterial community characterized by a decrease in *Lactobacillus* and an increase in anaerobic bacteria associated with bacterial vaginosis, such as *Gardnerella* [16]. This is consistent with other studies that show that persistent papillomavirus infection is associated with an increase in anaerobic bacteria [33,34]. Whether either member of this viral-bacterial correlate is causative, or whether each is simply present as a result of simultaneous exposure remains to be determined. The extension of this kind of analysis to disease etiologies and complex traits that are poorly understood may reveal new associations with viral infection and microbial community dynamics.

Virome analysis of the HMP and other data shows that more work is required to achieve an accurate understanding of the role of viruses in producing disease. The presence of certain viruses does not mean that there will invariably be clinical consequences. For example, we observed human adenovirus C in 17% of the nasal samples, yet these individuals did not have fever or acute illness. Likewise, in another study we found that 21% of afebrile children with minimal or no respiratory symptoms had rhinovirus present in nasopharyngeal secretions based on sequencing assays [35]. Taken together these data indicate that care is required in attributing disease manifestations to a virus that is found to be present, especially using sensitive molecular methods. We also detected adenovirus C in skin (retroauricular crease) samples from some healthy adults, which was unexpected because adenoviruses are typically associated with the respiratory or gastrointestinal tracts. Further work would need to be done to follow up this observation in a prospective study to determine whether the adenovirus sequences in skin samples represent infection or spread of virus-containing material from the respiratory tract. These early descriptions of the human virome suggest the need for additional studies to determine factors that distinguish asymptomatic infections from those that may explain a patient’s symptoms and require interventions including treatment with antiviral drugs.

The data presented here show that an extensive, unbiased analysis - such as that achieved with high-throughput deep metagenomic shotgun sequencing of a large number of human-derived samples from different body habitats - is a sound approach to identifying the scope of viruses that comprise the human virome, including novel viruses. There are a number of ways this analysis can be extended in the future. Including RNA viruses in the sequencing and analysis and expanding the body sites sampled would lead to a more comprehensive view of the diversity in the human virome. Also, the methods used for DNA preparation and sequencing were limited to detecting ssDNA viruses with dsDNA intermediates, so future studies should include methods to assess ssDNA viral genomes. Deeper sequencing would allow the more robust detection of relatively rare viral sequences, improving the sensitivity of the sequencing assay. Deeper sequencing or the addition of quantitative follow-up assays would also allow us to assess viral dynamics by determining if the same virus strain is present in multiple body sites from the same subject or at multiple time points. Increasing the number of subjects would be beneficial because, while this is a large data set, eukaryotic virus communities are very simple and varied. Thus, some of the results from this set are anecdotal and bring attention to questions that can be better examined in future targeted studies. Sampling the same subjects at many time points would lead to an even more comprehensive understanding of the dynamics of the human virome, which is of particular interest for chronic infections. This study does not distinguish active and latent infections, and future studies could concentrate on active infections by assessing cell-free virus particles. This analysis relies on identifying sequence similarity to known viruses, and sequences from unknown novel viruses with no sequence similarity to known viruses may be present but not analyzed. New computational methods will thus be required to address this shortcoming. High-throughput sequencing technologies have the potential to play important roles in diagnostic virology, and expanding our knowledge of the human virome is a necessary first step to interpreting results from diagnostic tests and applying that information for effective diagnosis and treatment.

## Conclusions

This study is the first to use high-throughput DNA sequencing to describe the diversity of eukaryotic DNA viruses in a large cohort of individuals who were sampled at a multiple body sites. This analysis demonstrates that there is a ‘normal viral flora’ in generally healthy, asymptomatic individuals. The normal flora includes viruses from seven families: *Herpesviridae*, *Polyomaviridae*, *Papillomaviridae*, *Adenoviridae, Anelloviridae, Parvoviridae,* and *Circoviridae.* Some viruses establish long-term infections that may be associated with increased risk or possibly with protection from disease. A better understanding of the composition and dynamics of the virome may hold important keys to human health.

## Methods

### Subjects and sequence data

We analyzed mammalian DNA virus sequences in metagenomic, whole genome shotgun sequence (WGS) data sets from 706 samples, which were produced on the Illumina platform (Illumina, Inc., San Diego, California, United States) by the HMP (Additional file 1: Table S1) [2]. Our analysis was limited to DNA viruses because RNA was not isolated from the samples in the HMP study. Furthermore, sequencing library construction protocols are designed for dsDNA; therefore, our analysis focused on dsDNA viruses and dsDNA replicative intermediates of ssDNA viruses. The sampled sites included in the WGS data set were nose (anterior nares), skin (retroauricular crease), mouth (buccal mucosa, tongue dorsum, subgingival plaque, supragingival plaque, and throat), vagina (primarily posterior fornix, but a few samples from vaginal introitus and mid-vagina were also included), and gut (stool). In some cases, multiple (usually two) visits from the same individual were included. Samples were collected according to standardized protocols [36]. Clinicians collected samples from each anatomic site using Catch-All™ Sample Collection Swabs, with the exception of the stool sample, which was self-collected. DNA was extracted from each sample using the MoBio PowerSoil DNA Isolation Kit (MoBio Laboratories, Carlsbad, California, United States). The Illumina GAIIX platform was used to generate 100 bp paired-end reads, with the target of generating 10 Gb sequence per sample. BMTagger was used to identify human sequences, which were subsequently removed from the data set. Duplicate, low quality, and low complexity sequences were also trimmed or removed.

Clinical data from this study were jointly produced by the Baylor College of Medicine and the Washington University School of Medicine. Sequencing data were produced by the Baylor College of Medicine Human Genome Sequencing Center, The Broad Institute, the Genome Institute at Washington University, and the J. Craig Venter Institute. The metadata were submitted by the EMMES Corporation, which serves as the clinical data collection site for the HMP. Only a subset of samples collected by the HMP was subjected to WGS. Subjects were sampled using a protocol that was approved by the Institutional Review Board for Baylor College of Medicine and the Human Research Subjects Protection Office at Washington University School of Medicine, and written informed consent was obtained from all subjects [2,37]. Before they were admitted to the study or sampled, subjects were required to meet an extensive set of clinical criteria, which established them as generally healthy adults. Importantly, entry criteria established that the subjects were not symptomatic for acute infections, had not been diagnosed with HPV infection within the last two years, and had not had any active genital herpes infection within the last two months (females) [37]. Subjects were sampled up to three times at the same body sites, with visits separated by 30 to 359 days. The WGS data include samples from 102 young adults (18 to 40 years old), of whom 46 were female. Sample collection was divide across two locales, with 46% collected at Baylor College of Medicine in Houston, Texas, and 54% at Washington University in St Louis. Our study population comprised 86% Caucasian, 6% Black, and 8% Asian participants.

### Identification of viral sequences

We began analysis with the human-screened, processed data sets provided by the HMP [2]. Viral sequences were identified based on similarity to virus reference genomes. We optimized the analysis pipeline for viral sequence detection by increasing the sensitivity of the alignments. This was done by allowing mismatches between the query and reference and by including both nucleotide and translated sequence alignments so sequences that were divergent from reference genomes would be detected. This analysis is an improved version of the pipeline we described previously [35]. A brief description of the pipeline follows. First, sequence reads were aligned against a virus reference database using a tool for nucleotide sequence alignment. In this version of the pipeline, a Real Time Genomics map (Real Time Genomics, Hamilton, New Zealand) was used to align sequences to the reference sequence database. The following parameters allowed us to identify sequences with nucleotide sequence similarity to viral reference sequences: −-repeat-freq 97% -e 10% –w 15 –n 255 –penalize-unknowns. The sequences in the reference sequence database included all of the sequences classified as viral in the National Center for Biotechnology Information Nucleotide database [38], which were found by using the search term ‘virus’. This included viral genomes and partial viral sequences. Next, sequences that were not aligned were subjected to translated sequence alignments to the same viral references, which were translated in six frames. This version of the pipeline used MBLASTX software (MulticoreWare Sunnyvale, California, United States) [39] with the following parameters: −m 30 –e 1e-02. After this initial screen to identify sequences with similarity to viral genomes, the subset of sequences identified was aligned to the larger nucleotide and translated amino acid sequence databases [40], which include entries from a more comprehensive set of organisms. Again, this version of the pipeline used Real Time Genomics mapping and MBLASTX with the parameters described above. Finally, sequences that unambiguously aligned to viral references in the larger databases were considered viral and included in the downstream analysis, and sequences that could not be clearly classified, such as repetitive sequences, were disregarded. This is a conservative method for identifying viral sequences within the samples. The number of sequences aligned to mammalian DNA virus genera are shown in Additional file 1: Table S2. Endogenous retroviruses integrated into the human genome were excluded from analysis because many endogenous retrovirus sequences were removed during the human screening step carried out by the HMP Consortium, and, therefore, the endogenous retrovirus sequence counts obtained from our pipeline are incomplete. Virus sequences were classified at the genus level, and species level classifications were determined after manual review.

### Correlation of viruses with bacterial communities

The characterization of bacterial communities by the HMP was used to correlate viral and bacterial community structure in the vaginal samples [2,41]. Relative abundance of the bacterial communities was calculated by taking the (depth of coverage × 100 Mb/number of covered bases). Several small, incomplete references, which included ribosomal sequences, had been included in the HMP reference database. These references were removed from the report by excluding references smaller than 100,000 bases in length in the final report of organisms present in the vaginal samples. Patient data were obtained through the Database of Genotypes and Phenotypes (study accession phs000228.v2.p1 [42]). Samples were clustered based on Bray-Curtis dissimilarity of the bacterial community structure and visualized using iTOL [43].

## Additional file

Additional file 1:
**Supplementary information. Table S1** Short read archive accession numbers and sample information. **Table S2** Viral genera detected. **Table S3** Percentages of individuals in whom each virus was detected. **Table S4** Stability of the virome over two evaluations. **Table S5** Stability of the virome over two evaluations.

## Abbreviations

bp: base pair
dsDNA: double-stranded DNA
HMP: Human Microbiome Project
HHV: human herpesvirus
HSV: herpes simplexvirus
HPy: human polyomavirus
HPV: human papillomavirus
ssDNA: single-stranded DNA
WGS: whole genome shotgun sequence
